# P61 Raising antimicrobial resistance awareness among postgraduate medicine: a teaching innovation during World Antibiotic Awareness Week

**DOI:** 10.1093/jacamr/dlag102.067

**Published:** 2026-06-26

**Authors:** Rasha Abdelsalam Elshenawy, Marvelle Brown

**Affiliations:** School of Health, Medicine and Life Sciences, University of Hertfordshire, College Lane, Hatfield AL10 9AB, UK; School of Health, Medicine and Life Sciences, University of Hertfordshire, College Lane, Hatfield AL10 9AB, UK

## Abstract

**Background:**

Antimicrobial resistance (AMR) is one of the most critical priorities in global public health, with the WHO calling for urgent, coordinated action worldwide. Developing an AMR-competent postgraduate workforce capable of translating knowledge into effective stewardship action is central to any sustainable response.^1^ World Antimicrobial Awareness Week (WAAW), held annually 18–24 November, led globally by WHO and nationally by the UK Health Security Agency (UKHSA) under ‘Act Now. Protect Our Present. Secure Our Future’, offers a strategic opportunity to embed AMR and stewardship education within postgraduate medical and public health curricula.^2,3^

**Objectives:**

To develop, implement, and evaluate a structured WAAW-aligned AMR/AMS educational initiative within postgraduate medicine and public health education, assessing its impact on student engagement, awareness, and AMR-focused research activity, as a replicable model for higher education institutions nationally and internationally.

**Methods:**

A structured AMR awareness session was delivered to 187 postgraduate public health students by an AMR/AMS global health policy expert and academic staff, timed to coincide with WAAW 2025 (18–24 November). The session incorporated national and global AMR/AMS guidelines, surveillance frameworks, and stewardship toolkits, with supplementary resources made available to support further independent learning. Student engagement was assessed through qualitative feedback, and outcomes were evaluated by monitoring AMR-related academic participation and comparing AMR-focused dissertation and project topic selection against the preceding student cohort, to assess the sustainable impact of this innovative teaching approach on academic practice.

**Results:**

Following the WAAW teaching intervention, a marked and sustained increase in student engagement with AMR and AMS content was observed across both semesters. Qualitative feedback indicated heightened awareness of AMR's global burden, greater confidence in applying stewardship frameworks, and a stronger sense of professional responsibility in addressing AMR across community and healthcare settings. Most notably, a significantly higher proportion of students in the 2024–2025 cohort selected AMR and AMS-related themes for their independent research projects and Master's dissertations compared to the preceding cohort. Research topics spanned national AMR action plan analyses across multiple low- and middle-income countries, including Nigeria, India, Bangladesh, and South Africa, as well as systematic reviews of stewardship interventions and investigations of digital health and AI applications in AMR surveillance. These outcomes collectively suggest that early, structured, campaign-aligned AMR exposure within the postgraduate curriculum meaningfully shapes students' academic trajectories and research priorities.

**Conclusions:**

Integrating WAAW into postgraduate medicine and public health education represents an innovative and impactful approach to AMR/AMS training. Aligning national and global health campaigns with curriculum delivery produces measurable improvements in student awareness, engagement, and research focus. Combining national and international frameworks, such as UKHSA and WHO recognized stewardship frameworks, research-led teaching practice, and global partnerships offers a replicable and scalable blueprint for postgraduate AMR education nationally and internationally. This model, rooted in the WHO's call to Act Now. Protect Our Present. Secure Our Future., demonstrates that education is the foundation of sustainable AMR action. This initiative calls upon higher education institutions worldwide to follow, transforming research into practice in the global fight against AMR.

## References

[1] WHO . Global Call to Action to Address Antimicrobial Resistance. 2025. https://www.who.int/publications/m/item/global-call-to-action-to-address-antimicrobial-resistance

[2] WHO . World Antimicrobial Awareness Week 2025, Act Now: Protect Our Present, Secure Our Future. 2025. https://www.who.int/campaigns/world-amr-awareness-week/2025 10.46234/ccdcw2025.240 PMC12647938 41312525

[3] UKHSA . World Antimicrobial Awareness Week (WAAW) and European Antibiotic Awareness Day (EAAD). Gov.uk. 2025. https://www.gov.uk/government/publications/european-antibiotic-awareness-day-resources-toolkit-for-healthcare-professionals-in-england/world-antimicrobial-awareness-week-waaw-and-european-antibiotic-awareness-day-eaad

